# Identification of novel common breast cancer risk variants at the 6q25 locus among Latinas

**DOI:** 10.1186/s13058-018-1085-9

**Published:** 2019-01-14

**Authors:** Joshua Hoffman, Laura Fejerman, Donglei Hu, Scott Huntsman, Min Li, Esther M. John, Gabriela Torres-Mejia, Larry Kushi, Yuan Chun Ding, Jeffrey Weitzel, Susan L. Neuhausen, Paul Lott, COLUMBUS Consortium, Magdalena Echeverry, Luis Carvajal-Carmona, Esteban Burchard, Celeste Eng, Jirong Long, Wei Zheng, Olufunmilayo Olopade, Dezheng Huo, Christopher Haiman, Elad Ziv

**Affiliations:** 1 Department of Epidemiology and Biostatistics, Institute of Human Genetics, University of California, San Francisco, San Francisco, CA USA; 2 Division of General Internal Medicine, Department of Medicine, Institute of Human Genetics, Helen Diller Family Comprehensive Cancer Center, University of California, San Francisco, Box 0320, San Francisco, CA 94143 USA; 3 Division of General Internal Medicine, Department of Medicine, University of California, San Francisco, San Francisco, CA USA; 4 Department of Medicine, Stanford University, Stanford, CA USA; 5 Stanford Cancer Institute, Stanford University, Stanford, CA USA; 6 National Institute of Public Health, Cuernavaca, Mexico; 7 Division of Research, Kaiser Permanente, Oakland, Northern California USA; 8 Beckman Research Institute of City of Hope, Department of Population Sciences, City of Hope, Duarte, CA USA; 9 City of Hope National Medical Center, Clinical Cancer Genetics, Duarte, CA USA; 10 Department of Biochemistry and Molecular Medicine, University of California, Davis, Davis, CA USA; 11 University of Tolima, Ibague, Colombia; 12 Department of Biophamaceutical Sciences, Lung Biology Center, University of California, San Francisco, San Francisco, CA USA; 13 Department of Medicine, Institute for Human Genetics, University of California, San Francisco, San Francisco, CA USA; 14 Department of Epidemiology, Vanderbilt University Medical Center, Nashville, TN USA; 15 Department of Public Health Sciences, University of Chicago School of Medicine, Chicago, IL USA; 16 Department of Medicine, Section of Oncology, University of Chicago School of Medicine, Chicago, IL USA; 17 Department of Preventive Medicine, Norris Comprehensive Cancer Center, Keck School of Medicine, University of Southern California, Los Angeles, CA USA

**Keywords:** Genome wide association study, Fine mapping, Hispanic/Latino populations

## Abstract

**Background:**

Breast cancer is a partially heritable trait and genome-wide association studies (GWAS) have identified over 180 common genetic variants associated with breast cancer. We have previously performed breast cancer GWAS in Latinas and identified a strongly protective single nucleotide polymorphism (SNP) at 6q25, with the protective minor allele originating from indigenous American ancestry. Here we report on fine mapping of the 6q25 locus in an expanded sample of Latinas.

**Methods:**

We performed GWAS in 2385 cases and 6416 controls who were either US Latinas or Mexican women. We replicated the top SNPs in 2412 cases and 1620 controls of US Latina, Mexican, and Colombian women. In addition, we validated the top novel variants in studies of African, Asian and European ancestry. In each dataset we used logistic regression models to test the association between SNPs and breast cancer risk and corrected for genetic ancestry using either principal components or genetic ancestry inferred from ancestry informative markers using a model-based approach.

**Results:**

We identified a novel set of SNPs at the 6q25 locus associated with genome-wide levels of significance (*p* = 3.3 × 10^− 8^ - 6.0 × 10^− 9^) not in linkage disequilibrium (LD) with variants previously reported at this locus. These SNPs were in high LD (*r*^2^ > 0.9) with each other, with the top SNP, rs3778609, associated with breast cancer with an odds ratio (OR) and 95% confidence interval (95% CI) of 0.76 (0.70–0.84). In a replication in women of Latin American origin, we also observed a consistent effect (OR 0.88; 95% CI 0.78–0.99; *p* = 0.037). We also performed a meta-analysis of these SNPs in East Asians, African ancestry and European ancestry populations and also observed a consistent effect (rs3778609, OR 0.95; 95% CI 0.91–0.97; *p* = 0.0017).

**Conclusion:**

Our study adds to evidence about the importance of the 6q25 locus for breast cancer susceptibility. Our finding also highlights the utility of performing additional searches for genetic variants for breast cancer in non-European populations.

**Electronic supplementary material:**

The online version of this article (10.1186/s13058-018-1085-9) contains supplementary material, which is available to authorized users.

## Introduction

Breast cancer is a partially heritable disease. Mutations in several high-penetrance genes including *BRCA1* [1, 2], *BRCA2* [3], and others [4] are associated with high risk of breast cancer among carriers and explain a fraction of the heritability. Genome-wide association studies (GWAS) have identified over 180 common single nucleotide polymorphisms (SNPs) associated with risk of breast cancer [5–20]. The majority of these SNPs were identified in European ancestry and East Asian ancestry populations, although some unique SNPs have been identified in African American populations [21] and in Latina populations [22, 23].

Several GWAS studies have identified SNPs at 6q25 that are associated with breast cancer risk [13, 18, 20, 23–27] and mammographic density [23, 27–30]. The initial report identified a SNP in the intergenic region between *ESR1* and *CDCC170* in an East Asian population [24]. The locus was then confirmed in other populations and several additional variants were identified [11, 18, 25, 26, 31]. More recently, a fine-mapping and functional approach at this locus identified five distinct common variants associated with risk of different subtypes of breast cancer [27].

Hispanic/Latino populations are the second largest ethnic group in the USA [32] and yet have been understudied in GWAS [33]. Latinos are a population of mixed ancestry with European, indigenous American and African ancestral contributions [34–37]. Since there are no large studies of breast cancer in indigenous American populations, studies in Latinos may identify novel variants associated with breast cancer that are unique to or substantially more common in this population. We have previously used an admixture mapping approach to search for breast cancer susceptibility loci in Latinas and identified a large region at 6q25 where indigenous American ancestry was associated with decreased risk of breast cancer [22]. Subsequently, we identified a SNP (rs140068132) that was common (minor allele frequency ~ 0.1) only in Latinas with indigenous American ancestry and was associated with substantially lower risk of breast cancer, particularly estrogen receptor (ER)-negative breast cancer, and with lower mammographic density [23]. However, the variant we identified did not completely explain the risk associated with locus-specific ancestry at 6q25 in Latinas, suggesting that other variants may account for this risk. We set out to fine-map and identify additional variants at 6q25 associated with breast cancer risk among Latinas.

## Methods

### Populations

San Francisco Bay Area breast cancer study (SFBCS): the SFBCS is a population-based multiethnic case–control study of breast cancer. Patients (cases) aged 35–79 years diagnosed with invasive breast cancer from 1995 to 2002 were identified through the Greater Bay Area Cancer Registry. Controls were identified by random-digit dialing and matched on 5-year age groups. Blood collection was initiated in 1999. For this study, we focused only on patients and matched controls who self-identified as Latina or Hispanic and included 351 cases and 579 controls. Samples from this study were used as part of the initial discovery set.

Breast Cancer Family Registry (BCFR): the BCFR is an international, National Cancer Institute (NCI)-funded family study that has recruited and followed over 13,000 breast cancer families and individuals with breast cancer with strong likelihood of genetic contribution to disease. The present study includes samples from the population-based Northern California site of the BCFR. Cases in patients aged 18–64 years diagnosed from 1995 to 2007 were ascertained through the Greater Bay Area Cancer Registry. Cases in patients with indicators of increased genetic susceptibility (diagnosis at the age of < 35 years, bilateral breast cancer with the first diagnosis at the age of < 50 years, a personal history of ovarian or childhood cancer, and a family history of breast or ovarian cancer in first-degree relatives) were oversampled. Cases not meeting these criteria were randomly sampled.

Population controls were identified through random-digit dialing and frequency-matched on 5-year age groups to cases diagnosed from 1995 to 1998. We included 641 cases and 61 controls who self-identified as Latina or Hispanic from this study. Samples from this study were used as part of the initial discovery set.

Since the SFBCS and BCFR were recruited from the same region and during an overlapping time frame, we combined these datasets to search for relatives. After removing relatives (preferentially keeping cases) and samples that overlapped with the Kaiser Research Project on Genes, Environment and Health, we included 942 cases and 589 controls from these studies.

Multiethnic cohort (MEC): the MEC is a large prospective cohort study in California (mainly Los Angeles County) and Hawaii. The breast cancer study is a nested case–control study including women with invasive breast cancer diagnosed at the age of > 45 years and controls matched on age (within 5 years) and self-identified ethnicity. After removing relatives (preferentially keeping cases), we used phenotypic and genetic data from 520 Latina breast cancer cases and 1544 matched Latina controls. Samples from this study were used as part of the initial discovery set.

Research project on genes environment and health (RPGEH): the RPGEH is a large cohort study of over 100,000 men and women of all racial/ethnic groups who are members of the Kaiser Permanente Health Plan. This analysis focuses only on women who are of self-reported Latina/Hispanic ethnicity (*N* = 3801). We included both incident and prevalent cases (total *N* = 225) in our analyses. We identified 44 women who were also included in the SFBCS. The genetic data from these participants were included as part of the RPGEH since we considered the Affymetrix Lat array as a more comprehensive array than the Affymetrix 6.0 array. After removing relatives, we included a total of 225 cases and 3574 controls. Samples from this study were used as part of the initial discovery set.

Cancer de mama (CAMA) study: this study is a population-based case–control study of breast cancer conducted in Mexico City, Monterrey, and Veracruz. Patients (cases) aged 35–69 years diagnosed between 2005 and 2007 were recruited from 11 hospitals (3–5 in each region). Controls were recruited based on membership in the same health plan as the cases and are frequency-matched on 5-year age groups. For the current study, we used phenotypic data and DNA samples from 1008 women with breast cancer and 1063 controls. Of these, 698 cases and 599 controls were genotyped with the Illumina Oncoarray and included in the discovery. An additional 310 cases and 464 controls were included as part of the replication dataset.

Colombian Study of Environmental and Heritable Causes of Breast Cancer (COLUMBUS): COLUMBUS is a population-based case–control study of breast cancer conducted in four cities: Bogota, Ibague and Neiva from the Central Colombian Andes region, and Pasto, from the Colombian South. Patients aged 18–75 years, with incident cases of invasive breast cancer, have been recruited in two population registries and two large cancer hospitals. Recruitment started in 2011. Cancer-free controls were recruited through the same institutions and were matched on education, socioeconomic status and local origin using a genealogical interview. In the current study, we used data from 954 cases and 769 controls for the replication study.

Hereditary Cancer Registry of City of Hope (HCRCOH) (Southern California; PI Jeffrey Weitzel): Latina breast cancer cases are part of the HCRCOH through the Clinical Cancer Genetics Community Research Network (CCGCRN). The CCGCRN includes cancer center and community-based clinics that provide genetic counseling to individuals with a personal or family history of cancer [38]. All patients are invited to participate in the HCRCOH at the time of consultation (> 90% participation). Starting in May 1998 and continuing to the present, women of self-reported Latina origin with breast cancer were seen for genetic counseling, were enrolled in the Registry and underwent *BRCA1/2* testing after providing informed consent. In the current study we genotyped 1148 cases. The 347 unaffected female Latina controls were from Southern California and were invited to participate at community health fairs, via flyers, and at City of Hope. These samples were used as part of the replication study.

African American breast cancer GWAS (AABC): the GWAS includes African American participants from nine epidemiological studies of breast cancer, comprising a total of 3153 cases and 2831 controls (cases/controls: the MEC, 734/1003; the Los Angeles component of the Women’s contraceptive and reproductive experiences (CARE) study, 380/224; the Women’s circle of health study (WCHS), 272/240; the SFBCS, 172/231; the Northern California Breast Cancer Family Registry (NC-BCFR), 440/53;the Carolina breast cancer study (CBCS), 656/608; The Prostate, Lung, Colorectal, and Ovarian Cancer Screening Trial (PLCO) Cohort, 64/133; the Nashville breast health study (NBHS), 310/186; and the Wake Forest University breast cancer study (WFBC), 125/153). Additional details have previously been reported [21, 39]. These samples were used as part of the replication study.

The ROOT consortium included six studies and a total of 1657 cases and 2029 controls of African ancestry: the Nigerian Breast Cancer Study (NBCS), 711/624; the Barbados national cancer study (BNCS), 92/229; the Racial variability in genotypic determinants of breast cancer risk study (RVGBC), 145/257; the Baltimore Breast cancer study (BBCS), 95/102; the Chicago cancer prone study (CCPS), 394/387; and the Southern community cohort (SCCS), 220/430. Additional details can be found elsewhere [21]. These samples were used as part of the replication study.

Shanghai breast cancer genetics study: study participants were drawn from four population-based studies conducted in Shanghai, the Shanghai Breast Cancer Study (SBCS), Shanghai Women’s Health Study (SWHS), Shanghai Breast Cancer Survival Study (SBCSS), and the Shanghai Endometrial Cancer Study (SECS (which contributed control data only). The SBCS is a population-based, case-control study conducted in urban Shanghai. Subject recruitment in the initial phase of the SBCS (SBCS-I) was conducted between August 1996 and March 1998. The second phase (SBCS-II) of recruitment occurred between April 2002 and February 2005. Breast cancer cases were identified through the population-based Shanghai Cancer Registry and supplemented by a rapid case-ascertainment system. Controls were randomly selected using the Shanghai Resident Registry. The SBCSS included newly diagnosed breast cancer cases ascertained via the Shanghai Cancer Registry between April 2002 and December 2006. The SECS is a population-based, case–control study of endometrial cancer conducted between January 1997 and December 2003 using a protocol similar to the SBCS; only community controls from the SECS were included in the present study. The SWHS is a population-based prospective cohort study of women recruited between 1996 and 2000. The cohort has been followed by a combination of record linkage and active follow up to identify cause-specific mortality and cancer incidence by sites. All these studies are conducted among Chinese women in Shanghai, using very similar protocols in data and sample collection. There were 2731 cases and 2135 controls genotyped with an Affymetrix 6.0 array and 1794 cases and 2059 controls genotyped with an Illumina MEGA array. These subsets were analyzed separately and included in a meta-analysis as part of the replication study.

European ancestry GWAS data: we also evaluated the top SNPs using summary statistics from a recent large GWAS of European-ancestry breast cancer cases and controls [40]. We downloaded the summary statistics the Breast Cancer Association Consortium (BCAC) website (http://bcac.ccge.medschl.cam.ac.uk/bcacdata/oncoarray/) and used the summary statistics from the combined analysis of individuals of European ancestry from the Oncoarray and iCOGS consortia.

### Genotyping

#### GWAS

The SFBCS and NC-BCFR samples were all genotyped with an Affymetrix 6.0 arrays at the University of California, San Francisco (UCSF). The MEC samples were genotyped with an Illumina 660 array at USC (520 Latina women with breast cancer and 546 matched Latina controls) and an additional 998 controls were typed on an Illumina 2.5 M array at the Broad Institute (Cambridge, MA, USA). The RPGEH samples were typed on an Affymetrix LAT array at UCSF. The CAMA samples were typed on an Ilumina Oncoarray at the Quebec Genome Center. The COLUMBUS samples were typed on an Affymetrix Biobank Array. Genotyping in the AABC consortium was conducted using the IlluminaHuman1M-Duo BeadChip. Genotyping in the ROOT consortium was conducted using the Illumina HumanOmni2.5-8v1 array at Johns Hopkins University Center for Inherited Disease Research. A subset of the Shanghai Breast Cancer Genetics Study (SBCGS) samples were typed on an Affymetrix 6.0 array. After quality control exclusions, the final data set included 2731 cases and 2135 controls. A second subset of the SBCGS were genotyped on an Illumina MEGA array. After quality control exclusions, the final data set included 1794 cases and 2059 controls. Data for four SNPs identified in the discovery stage were extracted from the SBCGS datasets and were included in the replication stage.

#### Replication genotyping

The CAMA samples and the CCGCRN samples, were genotyped using Taqman probes for rs3778609. The CAMA samples that were not included in the GWAS were genotyped at 106 ancestry informative markers from genotyped on a Sequenome platform as previously described [41]. CCGCRN samples included 100 ancestry informative markers that were included as part of a sequencing project. The sequence data were aligned to Human Genome Build 37 using Burrows-Wheeler alignment and genotype calls were made using Haplotypecaller, which is part of the GATK platform [42].

### Analysis

#### Genotyping quality control and imputation

Samples with > 5% missing genotypes were removed from each dataset. We dropped variants with > 5% missing data from each dataset. Since excess homozygosity is more common in populations with substructure, particularly with ancestry informative markers, we did not use deviation from Hardy-Weinberg equilibrium as a criterion for excluding markers. All datasets were entered mapped to Hg19. Each dataset was then phased using SHAPEIT and imputed using the Haplotype Reference Consortium (HRC) with Minimac3 [43]. For the MEC datasets that included both 660 K and 2.5 M arrays, we used the overlapping SNPs (*N* = 192,795) and imputed from those since we found that if imputing them separately and then analyzing them together produced a large number of false positives. Each of the remaining GWAS datasets were submitted to the HRC server individually for imputation. Only variants with imputation quality scores of *R*^2^ > 0.5 were selected for additional analysis. In a separate analysis, we imputed each of the datasets to the 1000 Genomes Reference Version 3 (October 2015 release) [44] with Minimac3.

Genotype imputation for the ROOT consortium was conducted using the *IMPUTE2* software [45] with the 1000 Genomes Project phase I cosmopolitan variant set as the reference panel (October 2011 release) [43]. Genotype imputation in AABC was conducted using *IMPUTE2* software [45] to a cosmopolitan panel of all 1000 Genome Project subjects (March 2012 release). Variants with imputation score > 0.3 were included in the analysis.

The Shanghai Breast Cancer Study GWAS data were phased with Minimac2 and imputed with SHAPEIT using 1000 Genomes Project phase 3. Only SNPs with a minor allele frequency (MAF) ≥ 0.01 and high imputation quality (RSQR ≥ 0.5) were included in the analyses.

We used KING [46] to identify relative pairs either within the RPGEH cohort or between the RPGEH and SFBCS and/or NC-BCFR and performed the same analysis within the MEC and the CAMA study. We identified pairs of individuals with kinship coefficient > 0.2 and dropped one from each of these pairs. If a relative pair included a case and control then we excluded the control. If a relative pair included two cases or two controls we randomly dropped one of them. We dropped 127 individuals to eliminate all closely related individuals from the combined RPGEH, SFBCS, and NC-BCFR.

#### Empirical assessment of imputation accuracy

We genotyped rs3778609, the top novel SNP, in the CAMA study in samples that also had GWAS data and checked the concordance between genotyped and imputed results.

#### Genetic ancestry inference

We implemented principal component (PC) analysis to assess genetic ancestry in each of the discovery datasets in unrelated individuals. To do so, we first LD-pruned typed SNPs with *r*^2^ > 0.2 in PLINK. With the remaining data, we determined the PCs using EIGENSTRAT [47] within smartpca. For the replication datasets, we used ancestry informative markers and used the program ADMIXTURE [48] to calculate genetic ancestry, assuming a three-population model with ancestry from African, European, and Native American populations. We also inferred genetic ancestry as derived from the program ADMIXTURE in the discovery GWAS dataset to perform sensitivity analyses.

#### Association testing

We performed single-variant association testing using logistic regression models and adjusting for PCs 1–10 in PLINK [49]. For the replication datasets we entered ancestry into the model as covariates. For discovery, we performed GWAS by study and then performed a fixed effects meta-analysis using METAL [50]. We also performed association testing separately for estrogen receptor (ER)-positive and ER-negative breast cancer using this approach. To calculate LD, we calculated *R*^2^ in the controls in our dataset using PLINK. We then performed conditional analyses by entering the most significant SNP in the model as a covariate in addition to PCs 1–10. We evaluated genome-wide inflation by estimating λ (λ ≤ 1.0 indicates no inflation). To test for heterogeneity with family history and study site we entered these as multiplicative interaction variables with the SNPs of interest in the logistic regression models and tested the significance of the interaction variables. To test for heterogeneity by age, we dichotomized at age 50 years and also tested for a multiplicative interaction with the SNPs. In addition, we re-tested the associations for the top SNPs adjusting for genetic ancestry from ADMIXTURE using logistic regression models. Heterogeneity analyses and the analysis of the top SNPs using ancestry estimates from ADMIXTURE were performed using Stata (Version 14).

#### Power

Based on the sample size for discovery (2385 cases and 6416 controls) we had ~ 80% power to detect an odds ratio of 1.25, 1.365, and 1.49 with allele frequencies of 0.4, 0.2, and 0.1 respectively.

#### Ranking SNPs by evidence for function

For each of the top index SNPs (rs140068132, rs851985, and rs3776809) we identified all of the other SNPs that they are in LD with (*R*^2^ > 0.5) and that have a *p* value that is at within 2 log (base 10) level of significance compared to the top SNP. We then entered each index SNP with the other SNPs from that cluster into regulomeDB [51], which uses Encode data to annotate SNPs and report on their likelihood of affecting gene expression. The level of evidence could include the SNP being in a DNAse hypersensitivity region and/or a region associated with transcription factor (TF) binding. Further weight is given if the SNP alters a TF binding motif.

## Results

### Individual association analyses

We conducted a meta-analysis across four GWAS discovery studies (Table 1) and identified 48 variants with genome-wide significant *p* values at the 6q25 susceptibility region (Additional file 1: Table S1). We saw no evidence for inflation of association statistics in genome-wide analysis (λ = 0.97). No additional genome-wide significant SNPs were identified.

**Table 1:** Discovery and replication samples used

Study	Genotyping platform	Cases	Controls
Discovery: Latinas			
BCFR/SFBACS	Affy 6.0	942	589
RPGEH	Affy Axiom	225	3574
MEC	Illumina 1 M, 2.5 M	520	1544
CAMA	Illumina Oncoarray	698	599
Total		2385	6416
Replication: Latinas			
COLUMBUS	Affy Axiom	954	769
CCGCRN	Taqman	1148	347
CAMA	Taqman	310	464
Total Latina replication		2412	1580
Replication: African American			
AABC	Illumina 1 M	3153	2831
ROOT	Illumina 2.5 M	1657	2029
Total African ancestry		4810	4860
Replication: East Asian ancestry			
Shanghai Breast Cancer Study	Affymetrix 6.0	2731	2135
Shanghai Breast Cancer Study	Illumina MEGA	1794	2059
Total East Asian population		4525	4194

**Fig. 1:**
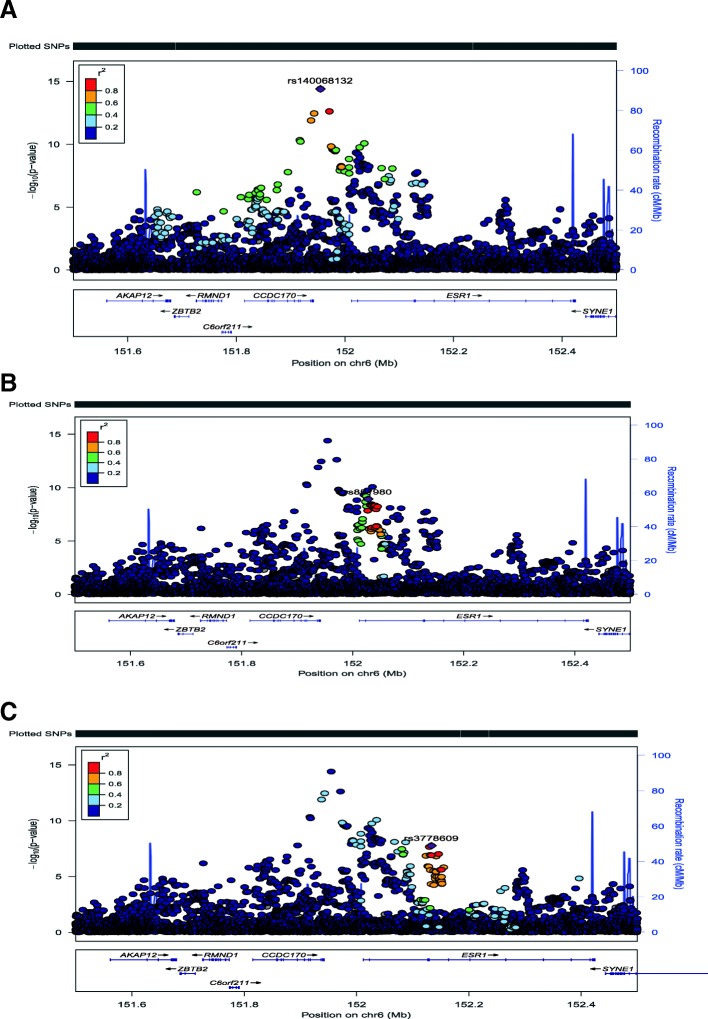
Association results with SNPs on chromosome 6 (chr6) from position 151.5 megabases (Mb) to 152.5 MB. On the x-axis are chromosome positions and on the y-axis are the negative log (base 10) *p* values. Each dot represents the meta-analysis results from the discovery genome-wide association study (GWAS) datasets. The different panels represent coloring by different linkage disequilibrium (LD) values related to an index SNP. Three index SNPs are selected including rs1400685132 (**a**), rs851980 (**b**), and rs3778609 (**c**)

Evaluating the SNPs at 6q25 and accounting for linkage disequilibrium (LD) between them, we found three distinct clusters of SNPs (haplotypes) associated at a genome-significant level (Fig. 1; Additional file 1: Table S1)

. The top variant in this region was rs140068132, which we previously reported as genome-wide significant in this population [23]. Of the remaining 47 top variants, 21 were in strong (*r*^2^ > 0.5) and another six were in moderate (0.39 < *r*^2^ < 0.49) LD with rs140068132, and none of these were significant in joint models with rs140068132 (Additional file 1: Table S1). Another set of 16 additional SNPs that was genome-wide significant, characterized by rs851980, were all in high LD (*r*^2^ > 0.5) with each other and in low LD (*r*^2^ < 0.2) with rs140068132. These SNPs include rs851984, which has been previously reported to be associated with breast cancer [27]. A third group of four SNPs at position ~ 151.13–152.15 megabases (Mb) on chromosome 6 (Hg19) was also genome-wide significant. These SNPs were characterized by rs3778609 and were in high LD (*r*^2^ > 0.9) with each and in low LD (*r*^2^ < 0.2) with rs140068132 or rs851985 and with other SNPs previously reported at this locus (Additional file 2: Table S2). The minor alleles of these SNPs are associated with lower risk of breast cancer, and the odds ratio (OR) for rs3778609 was 0.76 (95% CI 0.69–0.83; *p* = 6.0 × 10^− 9^; Table 2). These SNPs were well-imputed in each of the discovery GWAS with imputation *R*^2^ values > 0.95 for rs3778609 (Additional file 1: Table S1). Furthermore, in re-analysis of the top SNPs using 1000 Genomes as the reference dataset for imputation, we found a consistent genome-wide significant effect for these SNPs (Additional file 2: Table S3). We also evaluated the effect of controlling for genetic ancestry using a model-based approach (ADMIXTURE) in the discovery GWAS and found a consistent effect (Additional file 2: Table S4).

We performed conditional analyses by entering rs140068132 and other top SNPs at this locus in joint models. We found that rs3778609 remained nominally significant in a joint model adjusting for rs140068132 (Table 2), although the adjusted odds ratios were attenuated. We also found that rs851980 remained nominally significant in joint models with rs140068132 with mild attenuation. When we included three SNPs that best represent each of the signals from each set of associated variants (rs140068132, rs3778609, and rs851980) in the same model, all of the SNPs remained nominally significant with minimal attenuation of the odds ratios (compared to models including just pairs of variants; Table 2).

**Table 2:** Representative SNPs and association statistics from each of four different SNP clusters/regions that are genome-wide significant

SNP/risk allele	Allele frequency	Position (BP, Hg19)	Odds ratio (95% CI)	*P* value	Conditional OR* P value	Joint OR** P value
rs140068132-G	0.0933	6:151954834	0.58 (0.50–0.66)	4.4 × 10^− 15^		0.61 (0.53–0.71) 6.8 × 10^− 12^
rs851980-C	0.255	6:152027955	1.28 (1.18–1.35)	5.2 × 10^−10^	1.22 (1.12–1.32) 1.0 × 10^− 6^	1.19 (1.10–1.28) 3 × 10^− 6^
rs3778609-T	0.192	6:152133187	0.76 (0.69–0.83)	6.0 × 10^−9^	0.84 (0.76–0.93) 6.5 × 10^− 4^	0.86 (0.78–0.95) 0.0035

*Conditional on rs140068132

**Joint model with rs140068132, rs851985, and rs3778609

### Technical validation and replication

We evaluated the imputation accuracy of rs3778609 in a sample of 1369 women from the CAMA study, which had imputed genotypes from the GWAS and genotypes determined by Taqman assay. We found excellent agreement between the imputed and genotyped data with 1361/1369 (99.4%) of the genotypes being in concordance.

We used data from the portion of the CAMA study that did not have GWAS data, the COLUMBUS study and the CCGCRN to replicate the association with rs3778609. We found a consistent direction in all three studies and a nominally significant association in a meta-analysis of the three studies (*N* = 2412; *N* = 1620; OR = 0.88; 95% CI 0.78–0.99; *p* = 0.037, Table 3).

**Table 3:** Replication of rs3778609 in other Latina datasets

Study	Odds ratio (95% Cl)	*P* value
COLUMBUS	0.87(0.73–1.04)	0.119
CCGCRN	0.89(0.70–1.14)	0.375
CAMA (excluding GWAS)	0.88(0.69–1.13)	0.314
Meta-analysis	0.88(0.78–0.99)	0.037

*GWAS* genome-wide association studies

### Association of previously identified SNPs at 6q25

We examined previously reported SNPs in our discovery dataset (Additional file 2: Table S5). Only rs851984 was genome-wide significant in our study. Two other SNPs, rs3757322 and rs9397437, were nominally significant in the correct direction. The other two were not significantly associated with breast cancer in our study but the effects were directionally consistent with the previous reports and the 95% confidence intervals overlapped with the results from previous studies.

### Association with estrogen receptor subtypes and other sources of heterogeneity

We analyzed the association for each of the top SNPs separately and jointly by ER-status (Table 4). As we have previously reported, the minor (low risk) allele of rs140068132 is associated with a significantly (*p* = 0.04) lower odds ratio for ER-negative than for ER-positive breast cancer. We also found a significantly stronger (*p* = 0.025) effect size for ER-negative breast cancer for rs3778609. The effect size for rs851980 is also greater for ER-negative breast cancer; however, the difference between ER-negative and ER-positive breast cancer for this SNP was not statistically significant (*p* = 0.068).

**Table 4:** Association by estrogen receptor (ER) status

	ER-positive		ER-negative		*P* value for ER-positive vs. ER-negative
SNP	Odds ratio (95% CI)	*P* value	Odds ratio (95% CI)	*P* value	
rs140068132-G	0.59 (0.50–0.72)	2.7 × 10^−7^	0.39 (0.27–0.55)	2.0 × 10^− 7^	0.040
rs851980-C	1.19 (1.07–1.34)	0.002	1.46 (1.23–1.73)	1.3 × 10^−5^	0.068
rs3778609-T	0.74 (0.65–0.85)	1.1 × 10^− 5^	0.59 (0.47–0.73)	2.0 × 10^−6^	0.025

We also evaluated for other sources of heterogeneity for rs3778609 including location (San Francisco Bay Area versus Los Angeles versus Mexico), age (dichotomizing at age 50 years) and family history. We found no evidence of heterogeneity with study site (*p* = 0.13), age (*p* = 0.71), or family history (*p* = 0.61).

### Replication in non-Latinas

We evaluated the cluster of new SNPs we identified in this locus represented by rs37786109 in East Asians in the Shanghai breast cancer study and African ancestry populations in the ROOT and AABC studies and we also performed a look up of the effect in summary statistics from European populations (Table 5). We found a consistent effect for across these studies and a significant effect in a meta-analysis, although the odds ratio was a little attenuated compared to the odds ratio we observed in Latinas (Table 5).

**Table 5:** Associations of rs3778709 in non-Latina populations

Study	Population	MAF	OR	SE	*P* value
AABC	African American	0.304	0.93	0.044	0.10
ROOT	African, African American and West Indian	0.323	0.96	0.052	0.48
Shanghai 1	East Asian	0.265	0.91	0.048	0.065
Shanghai 2	East Asian	0.264	0.99	0.052	0.84
European (Oncoarray + iCOGs)	European and European American	0.016	0.94	0.025	0.025
Meta-analysis			0.95	0.017	0.0019

*MAF* minor allele frequency

### Evaluation of potential functional SNPs

We evaluated the top SNPs from each of the three clusters of genome-wide significant SNPs at 6q25 to determine which has the strongest evidence of potentially functional effects on gene expression using regulomeDB [51]. Among the SNPs in LD with rs3778609, rs6914438 and rs2071454 had the best evidence of a functional effect (Additional file 3: Table S6). Rs691443 had positive results in MCF-7 cells for both DNAse hypersensitivity and transcription factor binding. Rs2071454 is in the 5’ UTR of *ESR1* and is also annotated as being in a DNAse hypersensitive region and a transcription factor binding site. At the two previously reported loci, rs140068132 and rs851984 had the best evidence for functional effect in regulomeDB.

## Discussion

We have previously reported on a SNP at 6q25 associated with a minor allele that is unique to indigenous American populations and associated with decreased risk of breast cancer [23]. Here, we investigate this locus in greater depth in an expanded sample size of Latina breast cancer cases and controls. We have identified several SNPs that are genome-wide significant and associated with breast cancer at this locus independently of other SNPs previously reported by us in Latinas [23] and in other populations [11, 13, 24–27, 29, 31, 52, 53] at this locus. These SNPs are located in the region of ~  152.13–152.15 Mb (Hg19). Replication in African American, Asian, and European samples supports the association with these SNPs. In addition, we have also shown that these novel variants at this locus have significantly stronger effect sizes on ER-negative breast cancer. Prior studies have also demonstrated a stronger effect size with ER-negative breast cancer for most variants at the 6q25 locus, consistent with our data [18, 27].

Prior studies in other populations have reported a series of independent SNPs affecting breast cancer risk [11, 18, 24–27, 31]. A comprehensive fine mapping project using European and Asian ancestry populations identified five different clusters of SNPs at this locus [27]. We found that one of these SNPs, rs851984, was also genome-wide significant in Latinas, two more were nominally significant, and the remaining two were non-significant but had effects that were directionally consistent with those observed by Dunning et al.

Dunning et al. found that the variants they identified affect expression of *ESR1*, *RMND1* and *CCDC170* [27]. The *ESR1* gene encodes estrogen receptor alpha, a strong candidate gene for an effect on breast cancer risk. The effect we detected was stronger for ER-negative breast cancer, consistent with the effect of most of the other SNPs reported at this locus. This differential effect is unexpected since estrogen signaling via ER is believed to increase the risk of ER-positive breast cancer and not have an effect on ER-negative breast cancer [54, 55]. However, *ESR1* could also exert an effect via the stroma and many of the variants at this locus are known to affect mammographic density [27–30] which is associated with both ER-positive and ER-negative breast cancer [56]. Furthermore, other genes at this locus including *ARMT1, CCDC170*, and *RMND1* may also be candidates for an effect on breast cancer risk [27, 57–59]. *ARMT1* is protein methyl transferase that modifies DNA damage response [60] and small interfering RNA (siRNA) inhibition of *ARMT1* by siRNA suppresses proliferation of MCF-7 cells [59]. *CCDC170* is part of a recurrent rearrangement with *ESR1* found in aggressive ER-positive breast cancers [61]. This rearrangement leads to a truncated CCDC170 protein which, when introduced into ER+ breast cancer cells, leads to increased cell motility, anchorage-independent growth, reduced endocrine sensitivity, and enhanced xenograft tumor formation. Allele-specific expression studies suggest that breast cancer SNPs are more strongly associated with CCDC170 expression [57]. Since the new variants we report are common only in non-European ancestry populations, there are limited data to explore the potential effects of these variants on gene expression.

Our study is limited by sample size. Therefore, it is possible that we have missed other variants at this locus. Our study may also be affected by population stratification, which is more likely to occur in an admixed population such as Latinos. To reduce this problem, we adjusted for genetic ancestry in both the discovery and replication datasets. We found no evidence of genome-wide inflation of the association statistics in the discovery datasets. However, the replication datasets had fewer variants for the genetic ancestry estimates, which will lead to some noise in the ancestry estimate and may lead to insufficient adjustment. The studies we used included both population based and clinic-based recruitment and used different age ranges and, in some cases, family history criteria for ascertainment. Although we did not detect any interactions between the top new SNPs that we report and these factors, we may be underpowered to detect interactions.

We used the Haplotype Reference Consortium (HRC) for imputation, which includes a large number of reference individuals; however, the proportion of individuals with indigenous American ancestry in the HRC is low, and therefore, we are likely underpowered to detect rare variants that originate from American ancestry. The common variants that we report here are in the range that imputation from HRC is known to be relatively high quality. Furthermore, we validated our imputed results in one of the datasets by genotyping and found excellent correlation. Thus, we believe the new SNPs we report are based on relatively accurate imputation. However, we are likely missing some variants, particularly low-frequency variants from indigenous American origin. As imputation panels improve, we will be able to impute lower-frequency variants with more accuracy and may be able to detect new associations.

The effect size we observed in the replication dataset is substantially lower than in the discovery dataset, likely due to regression towards the mean, a well-known phenomenon in GWAS, since the top SNPs in a discovery dataset are likely to have some upward bias in the effect size [62]. However, even if we take the replication odds ratio (0.88) as the closest to the true effect size of these SNPs, this is still a relatively large effect for a common variant. We also found a consistent (though further attenuated) effect in populations of African, Asian, and European ancestry. The attenuated effect size in these populations may be due to different LD patterns or may be due to other non-genetic modifiers. Of note, despite the very large sample size used for discovery in Europeans, this variant had not reached genome-wide significance due to the low allele frequency in Europeans. This highlights the utility of using different ancestry populations including Latinas, to help discover new variants. It is likely that there are other variants that have not yet been identified in European GWAS due to low allele frequency and that could be identified in Latinas where they are more common. Larger studies of Latina women are needed to identify these variants.

## Conclusion

Our study demonstrates additional unique associations with variants at 6q25 and breast cancer risk. This further highlights the important contribution of this locus to breast cancer susceptibility, particularly ER-negative breast cancer susceptibility. Additional fine-mapping and functional studies are needed to elucidate all of the causal variants in our population. However, the variants we identified in this study can be useful to add to the increasing pool of common variants coming from GWAS and will be particularly useful to risk-stratify women of Latin American ancestry for breast cancer risk.

## Additional files

Additional file 1: **Table S1.** Association statistics and imputation accuracy for all SNPs with *p* value <5 × 10–7 in discovery dataset. Also included are results for models that are conditional on top SNPs and the and linkage disequilibrium between index SNPs described in the manuscript and other SNPs in this list. (XLSX 29 kb)

Additional file 2: **Tables S2**-**S5.** Supplementary tables including linkage disequilibrium between top SNPs and previously described SNPs at this locus. Association results for top SNPs with imputation to 1000 Genomes. Association results for top SNPs adjusted for ancestry using ADMIXTURE results. Association results for SNPs previously reported as genome wide significant at 6q25. (DOCX 27 kb)

Additional file 3: **Table S6.** Results of the top candidate SNPs analyzed using RegulomeDB (XLSX 12 kb)

## Abbreviations

AABC: African American breast cancer study
BCFR: Breast Cancer Family Registry
CAMA: Cancer de mama
CARE: Women’s contraceptive and reproductive experiences
CBCS: Carolina breast cancer study
CCGCRN: Clinical Cancer Genetics Community Research Network
CI: Confidence interval
COLUMBUS: Colombian study of environmental and heritable causes of breast cancer
ER: Estrogen receptor
GWAS: Genome-wide association study
HCRCOH: Hereditary cancer registry of City of Hope
IRB: Institutional review board
LD: Linkage disequilibrium
MAF: Minor allele frequency
Mb: Megabases
MEC: Multiethnic cohort
NC-BCFR: Northern California Breast Cancer Family Registry
OR: Odds ratio
PC: Principal component
PCA: Principal components analysis
RPGEH: Research project on genes environment and health
SBCS: Shanghai breast cancer study
SBCSS: Shanghai breast cancer survival study
SCCS: Southern community cohort study
SFBCS: San Francisco Bay Area breast cancer study
SNP: Single nucleotide polymorphism
SWHS: Shanghai women’s health study
UTR: Untranslated region
WCHS: Women’s circle of health study
