# Supplementary material for: Identification of novel common breast cancer risk variants at the 6q25 locus among Latinas
Source: Breast Cancer Res. 2019 Jan 14;21:3. doi: 10.1186/s13058-018-1085-9 (PMC6332913; doi:10.1186/s13058-018-1085-9)
Supplement: Supplementary file 2 — Tables S2-S5. Supplementary tables including linkage disequilibrium between top SNPs and previously described SNPs at this locus. Association results for top SNPs with imputation to 1000 Genomes. Association results for top SNPs adjusted for ancestry using ADMIXTURE results. Association results for SNPs previously reported as genome wide significant at 6q25. (DOCX 27 kb) [file 13058_2018_1085_MOESM2_ESM.docx]

Table S2: R^2^ with 6q25 SNPs reported by Dunning *et al.* [27]

| SNP | rs3757322 | rs9397437 | rs851984 | rs9918437 | rs2747652 |
| --- | --- | --- | --- | --- | --- |
| rs140068132 | 0.05 | 0.01 | 0.05 | 0 | 0 |
| rs851980 | 0 | 0.01 | **0.61** | 0.09 | 0 |
| **rs3778609** | 0.02 | 0 | 0.08 | 0.01 | 0 |

Table S3: Results of the top SNPs using 1000 Genomes as imputation reference

| **SNP/Risk allele** | **Allele Frequency** | **Odds Ratio**  **(95% CI)** | **P value** |
| --- | --- | --- | --- |
| rs140068132-G | 0.0909 | 0.56 (0.48-0.64) | 2.1x10^-15^ |
| RS851980-C | 0.255 | 1.27 (1.18-1.37) | 1.4x10^-9^ |
| rs3778609-T | 0.192 | 0.76 (0.69-0.83) | 7.4x10^-9^ |

Table S4: Results of the top SNPs adjusting for genetic ancestry using ADMIXTURE

| **SNP/Risk allele** | **Odds Ratio**  **(95% CI)** | **P value** |
| --- | --- | --- |
| rs140068132-G | 0.58 (0.51-0.66) | 4.0x10^-15^ |
| RS851980-C | 1.26 (1.17-1.35) | 3.3x10^-9^ |
| rs3778609-T | 0.77 (0.70-0.84) | 2.2x10^-8^ |

Table S5: Comparison of results from Latina GWAS with previously reported SNPs by Dunning *et al.* [27]

|  |  | **Results from European and Asian Fine Mapping from Dunning et al [27]** | | **Results from Latinas** | | |
| --- | --- | --- | --- | --- | --- | --- |
| SNP ID | BP_Allele | **Allele**  **Freq** | **Odds Ratio (95%CI)** | **Allele**  **Freq** | **Odds Ratio (95%CI)** | **P value** |
| rs3757322 | 151942194 | 0.33 | 1.09 (1.07-1.11) | 0.26 | 1.15 (1.05 – 1.26) | 0.001 |
| rs9397437 | 151952232 | 0.07 | 1.20 (1.16-1.24) | 0.06 | 1.26 (1.06 – 1.49) | 0.004 |
| rs851984 | 152023191 | 0.41 | 1.05 (1.03-1.07) | 0.35 | 1.25 (1.16 – 1.34) | 1.6x10^-9^ |
| rs9918437 | 152072718 | 0.07 | 1.10(1.06,1.14) | 0.09 | 1.07 (0.93 – 1.24) | 0.17 |
| rs2747652 | 152437016 | 0.54 | 1.07(1.05,1.09) | 0.50 | 1.04 (0.95 – 1.17) | 0.11 |
